# AP-2α regulates migration of GN-11 neurons via a specific genetic programme involving the Axl receptor tyrosine kinase

**DOI:** 10.1186/1741-7007-7-25

**Published:** 2009-05-22

**Authors:** Francesca Orso, Richard Jäger, Raffaele Adolfo Calogero, Hubert Schorle, Piero Sismondi, Michele De Bortoli, Daniela Taverna

**Affiliations:** 1 Molecular Biotechnology Center, University of Torino, via Nizza, 52, 10126, Torino, Italy; 2 Department of Oncological Sciences, University of Torino, SP142, 10060, Candiolo, Italy; 3 Institute of Pathology, Department of Developmental Pathology, University of Bonn Medical School, Sigmund-Freud-Strasse 25, 53127, Bonn, Germany; 4 Bioinformatics and Genomics Unit, Department of Clinical and Biological Sciences, University of Torino, Regione Gonzole 10, Orbassano, 10043, Torino, Italy; 5 Institute for Cancer Research and Treatment (IRCC), SP 142, 10060 Candiolo (To), Italy; 6 Center for Complex Systems in Molecular Biology and Medicine, University of Torino, Torino, Italy

## Abstract

**Background:**

Neuronal migration is a crucial process that allows neurons to reach their correct target location to allow the nervous system to function properly. AP-2α is a transcription factor essential for neural crest cell migration and its mutation results in apoptosis within this cell population, as demonstrated by genetic models.

**Results:**

We down-modulated AP-2α expression in GN-11 neurons by RNA interference and observe reduced neuron migration following the activation of a specific genetic programme including the Adhesion Related Kinase (*Axl*) gene. We prove that *Axl *is able to coordinate migration per se and by ChIP and promoter analysis we observe that its transcription is directly driven by AP-2α via the binding to one or more functional AP-2α binding sites present in its regulatory region. Analysis of migration in AP-2α null mouse embryo fibroblasts also reveals an essential role for AP-2α in cell movement via the activation of a distinct genetic programme.

**Conclusion:**

We show that AP-2α plays an essential role in cell movement via the activation of cell-specific genetic programmes. Moreover, we demonstrate that the AP-2α regulated gene *Axl *is an essential player in GN-11 neuron migration.

## Background

Neuronal migration is a crucial process that allows neurons to reach their correct target location from the site of origin. It takes place mainly during the embryonic period but a significant number of neurons migrate after birth, well into adulthood. Appropriate migration is essential for the construction of functional synaptic circuitries in the brain. A classical example is the migration of neurons related to sexual behaviours, such as the gonadotropin releasing hormone (GnRH^+^) neurons [1], through the olfactory compartment and into the hypothalamus. GnRH^+ ^neurons can be analysed *in vitro *using the immortalized and highly motile murine GN-11 cell line, which represents an ideal model to study the molecular bases of neuronal migration.

The AP-2α transcription factor is abundant in GN-11 cells and it is known to play a major role in controlling neuronal gene expression and nervous system development as demonstrated by several investigations [2,3]. In particular, genetic models such as zebrafish and mouse [4-7] showed that AP-2α is essential for neural crest cell migration and its mutation results in apoptosis within this cell population. AP-2α is a member of the AP-2 family which consists of five closely related proteins of Mr 50,000, AP-2α, β, γ, δ and ε (see [8-10]) encoded by distinct genes. These transcription factors can form homodimers or heterodimers via helix-span-helix motifs and transactivate their target genes by binding to GC-rich consensus sequences in the promoter regions [11]. Dopamine β-hydroxylase [12], human proenkephalin [13], acetylcholinesterase [14], rat luteinizing hormone receptor (LHRH) [15] and neuropeptide Y receptor [16] are key genes for central nervous system biology which are known to be transcriptionally regulated by AP-2 proteins. In addition, AP-2 proteins regulate genes involved in certain neuropathologies, such as the presenilin-1 gene, involved in Alzheimer's disease [17] and the huntingtin gene, involved in Huntington's disease [18]. A role for AP-2α protein in the biology of LHRH neurons has been demonstrated *in vivo *[19]; in fact, LHRH expression was greatly decreased in AP-2α knock-out mice compared with controls. However, the mechanism leading to the inhibition of LHRH expression was not elucidated since a direct regulation for AP-2α on the LHRH promoter was not demonstrated.

Here we present data showing the importance of AP-2α in the transcriptional regulation of genes that coordinate GN-11 neuron migration. We knocked down AP-2α expression by RNA interference (RNAi) and analysed migration and motility. We performed a microarray analysis to identify the genetic programme activated by AP-2α and observed the modulation of a master regulator of GnRH^+ ^neuron migration, the Adhesion Related Kinase (*Ark*) also called *Axl*. We proved that Axl is essential for GN-11 cell movement and that AP-2α directly regulates *Axl *transcription by binding to canonical AP-2 binding sites present in the promoter of this gene. In addition, we present data demonstrating an essential role for AP-2α in mouse embryo fibroblast migration via the modulation of a distinct set of genes compared with GN-11 cells.

## Results

### Production of stable AP-2α knock-down GN-11 cells

**Figure 1:**
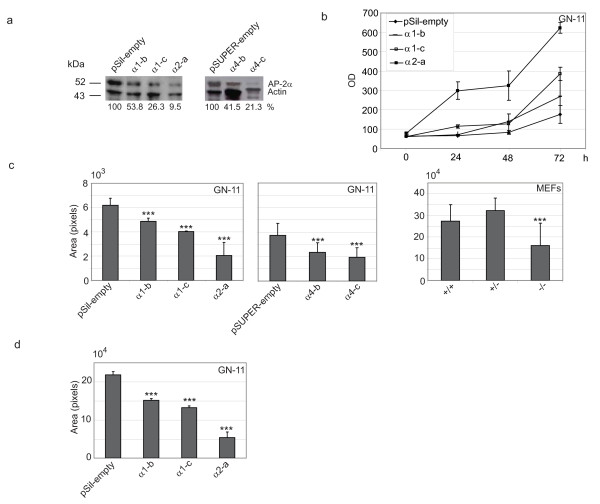
**AP-2α-dependent proliferation, migration and motility in GN-11 neurons and mouse embryo fibroblasts**. (a) Stable GN-11 control clones named pSil-empty and pSUPER-empty or AP-2α-silenced clones named α1-a, α1-c, α2-a, α4-b and α4-c were analysed for their AP-2α protein levels by Western blot (WB). mAb 3B5 was used to detect AP-2α protein expression and actin expression was evaluated as protein loading control. The percentage values correspond to the amount of AP-2α protein present in each clone. (b) Proliferation was analysed in control pSil-empty or AP-2α-silenced α1-b, α1-c, α2-a GN-11 clones. Cells were plated and starved for 24 hours in serum-free medium, then 10% foetal calf serum (FCS) was added to cells. Cells were fixed and stained at the indicated time and optical density was measured. The experiments were performed in triplicate and repeated twice. (c) Migration was analysed in transwell assays for pSil-empty or pSUPER-empty or AP-2α-silenced α1-b, α1-c, α2-a, α4-b and α4-c GN-11 clones as well as for three independent preparations of AP-2α +/+, +/- and -/- mouse embryo fibroblasts (MEFs). Cells were plated in serum-free medium in the upper chamber and allowed to migrate over the medium containing 10% FCS for 18 hours. Each experiment was performed in triplicate and repeated twice. (d) pSil-empty or α1-b, α1-c, α2-a GN-11 clones were used to analyse motility in wound healing assays. Cells were grown at 90% confluency, serum starved for 24 hours, then a wound (cross) was made in the cell layer. 10% FCS medium was added and cells were allowed to migrate for 18 hours. Pictures of the right arm of the cross were taken at *t *= 0 hours and at *t *= 24 hours. Quantitations were performed as described in [24]. Differences were statistically significant as measured by a two-tailed Student's *t*-test (***, *p *< 0.05). (In (b), (c) and (d) the bars represent ± standard deviations.)

GN-11 neurons were manipulated by RNAi to down-regulate AP-2α, the only AP-2 family member that they express (data not shown). Four different siRNA sequences were cloned in either pSilencer 1.0-U6 or pSUPERretro.puro expression vectors and expressed in GN-11 neurons following transfections and puromycin selection. The pIRES.puro2 selection plasmid was cotransfected with the pSilencer 1.0-U6-based vectors. After selection 10 empty control clones (five containing the pSilencer 1.0-U6; five containing the pSUPERretro.puro) as well as 26 clones expressing shRNA-α1, shRNA-α2, shRNA-α3 or shRNA-α4 (six, seven, seven and six clones, respectively) were isolated. Each clone was analysed for vector integration in the genome by polymerase chain reaction (PCR; data not shown). Silencing was verified by Western blot and quantitative real-time polymerase chain reaction (qRT-PCR) for all clones (data not shown). Clones α1-b, α1-c, α2-a, α4-b and α4-c showed the best AP-2α down-modulation compared with controls (Figure 1a) and were further used for biological assays and gene expression profiling.

### AP-2α modulates proliferation or migration in GN-11 neurons or mouse embryo fibroblasts

Analysis of cell proliferation was performed for pSil-empty or α1-b, α1-c, α2-a GN-11 clones. Two of the three analysed clones showed increased proliferation rate at 72 hours, proportional to the level of AP-2α expression (Figure 1b). In particular, the α2-a clone, which expressed the lowest level of AP-2α, showed a 3-fold increase in proliferation, 72 hours after starvation, suggesting that AP-2α controls GN-11 cell proliferation in a negative way. To understand the role of AP-2α in cell movement we compared the migration of control pSil-empty or pSUPER-empty clones with low AP-2α-expressing clones in response to 10% foetal calf serum (FCS) by transwell assays. As shown in Figure 1c reduced migration was observed for all AP-2α silenced clones, in particular α2-a and α4-c clones showed a 3-fold and a 1.5-fold reduction, respectively. Slighter reductions were observed for the other clones. A similar reduction in migration was observed when comparing AP-2α null versus wild-type or heterozygous mouse embryo fibroblasts (MEFs) (Figure 1c). Motility was monitored in GN-11 neurons for control pSil-empty and α1-b, α1-c and α2-a clones by wound healing assays (Figure 1d). After wounding, cells were allowed to migrate for 18 hours to recover the wound. A 40–80% reduction in motility was observed in AP-2α low-expressing clones compared with control cells. In particular, the α2-a clone showed an 80% decrease in motility, while a slighter decrease was observed for the other clones.

### Identification of potential AP-2α regulated genes by microarray analysis

**Figure 2:**
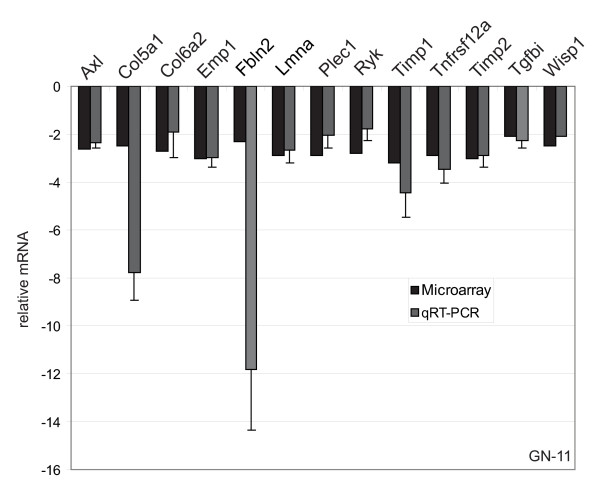
**Microarray analysis validation for GN-11 neurons**. Microarray data (Table 1 and Additional file 1) were validated by quantitative real-time polymerase chain reaction (qRT-PCR) performed in triplicate for 13 genes on two different RNA preparations from control pSUPER-empty or AP-2α low-expressing (α4-c) clones. The 18S or GAPDH housekeeping gene was used as an internal control to normalize the data. Microarray analysis and qRT-PCR fold changes are shown for each validated gene as average values. Bars represent ± standard deviations.

**Figure 3:**
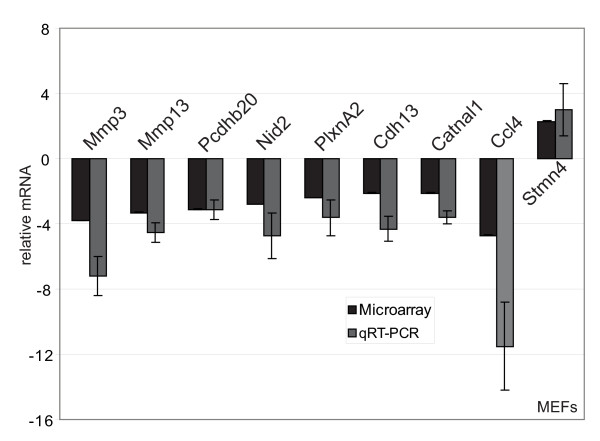
**Microarray analysis validation for mouse embryo fibroblasts (MEFs)**. Microarray data (Additional file 2) were validated by quantitative real-time polymerase chain reaction (qRT-PCR) for nine genes on RNA extracted from three independent AP-2α +/- or -/- mouse embryo fibroblast (MEF) preparations. The 18S rRNA or GAPDH housekeeping genes were used as internal controls to normalize the data. qRT-PCRs were performed in triplicate on all different RNA preparations. Microarray analysis and qRT-PCR fold changes are shown for each validated gene as average values. Bars represent ± standard deviations.

In order to identify which AP-2α regulated genes were involved in GN-11 neuron or MEF migration, microarray analyses were performed. For GN-11 cells total RNA was extracted from pSUPER-empty and α4-c clones and subjected to transcriptome analysis on Illumina Ref-8 BeadChips. Differential gene expression analysis revealed 510 modulated transcripts (305 decreased and 205 increased) in AP-2α silenced cells compared with controls (see Methods). A complete list of genes is shown in Additional file 1. A partial list of the most relevant genes is shown in Table 1, where genes are distributed in different Gene Ontology (GO) categories, such as cell cycle (i.e. *Ccnd1*, *Cdk5rap3 *and *Cetn3*), apoptosis (i.e. *Siva*, *Birc5 *and *Tnfrsf12a*), development (i.e. *Emp1*, *Wisp1*, *Ryk*), extracellular matrix (ECM)/cell adhesion (i.e. *Col5a1*, *Col6a1*, *Mfge8*) and cell migration (i.e. *Axl*, *Rtn4*, *Nup62*). Microarray data were validated by qRT-PCR for 13 genes shown to be involved in migration from the literature with a fold change greater than 2.0 (*Axl*, *Col5a1*, *Col6a2*, *Emp1*, *Fbln2*, *Lmna*, *Plec1*, *Ryk*, *Timp1*, *Tnfrsf12a*, *Timp2*, *Tgfbi*, *Wisp1*) on two different RNA preparations (Figure 2). Data were normalized using the 18S or GAPDH gene as internal controls. Gene expression was analysed in AP-2α +/- and -/- MEFs on Agilent Whole Mouse Genome 44 K platform and 1492 modulated transcripts (878 decreased and 614 increased) were found (Additional file 2). These results were validated by qRT-PCR for nine modulated genes randomly chosen (*Mmp3*, *Mmp13*, *Pcdhb20*, *Nid2*, *PlxnA2*, *Cdh13*, *Catnal1*, *Ccl4*, *Stmn4*) on three different RNA preparations (Figure 3). Also in this case the 18S or GAPDH housekeeping gene was used for qRT-PCR normalization data. An overlapping group of 26 genes was identified comparing the two datasets (Table 2), suggesting that some common mechanisms could be present in the different cellular systems but that most of the AP-2α-driven genetic programmes are cell-specific.

**Table 1:** Differentially expressed genes in AP-2α low-expressing GN-11 clones

**Gene**	**Description**	**Accession number**	**FC**
**Cell cycle**			
Ccnd1	cyclin D1 (Ccnd1), mRNA.	NM_007631	-3.0
Cetn3	centrin 3 (Cetn3), mRNA.	NM_007684	-2.7
Cdc2a	cell division cycle 2 homolog A (S. pombe)	NM_007659	-2.4
Cdkn2a	cyclin-dependent kinase inhibitor 2A (Cdkn2a), mRNA.	NM_009877	-2.4
Skp2	S-phase kinase-associated protein 2 (p45)	NM_013787	-2.2
Kras2	Kirsten rat sarcoma oncogene 2, expressed (Kras2), mRNA.	NM_021284	-2.1
Pa2g4	proliferation-associated 2G4 (Pa2g4), mRNA.	NM_011119	2.2
Stmn1	stathmin 1 (Stmn1), mRNA.	NM_019641	2.3
Tgif	TG interacting factor (Tgif), mRNA.	NM_009372	2.4
Anapc1	anaphase promoting complex subunit 1 (Anapc1), mRNA.	NM_008569	2.5
Atf5	activating transcription factor 5 (Atf5), mRNA.	NM_030693	2.5
Cdk5rap3	CDK5 regulatory subunit associated protein 3 (Cdk5rap3), mRNA.	NM_030248	2.8

**Apoptosis**			
Tnfrsf12a	tumor necrosis factor receptor superfamily, member 12a (Tnfrsf12a), mRNA.	NM_013749	-2.8
Ngfrap1	nerve growth factor receptor (TNFRSF16) associated protein 1 (Ngfrap1), mRNA.	NM_009750	-2.7
Ppm1f	protein phosphatase 1F (PP2C domain containing) (Ppm1f), mRNA.	NM_176833	-2.7
Gpx1	glutathione peroxidase 1 (Gpx1), mRNA.	NM_008160	-2.7
Siva	Cd27 binding protein (Hindu God of destruction) (Siva), mRNA.	NM_013929	-2.6
Birc5	baculoviral IAP repeat-containing 5 (Birc5), mRNA.	NM_009689	-2.6
Prkar1a	protein kinase, cAMP dependent regulatory, type I, alpha (Prkar1a), mRNA.	NM_021880	-2.5
Cycs	cytochrome c, somatic (Cycs), mRNA.	NM_007808	-2.5
Syvn1	synovial apoptosis inhibitor 1,	NM_028769	2.5
Sqstm1	sequestosome 1 (Sqstm1), mRNA.	NM_011018	2.9

**Development**			
Emp1	epithelial membrane protein 1 (Emp1), mRNA.	NM_010128	-3.0
Csrp2	cysteine and glycine-rich protein 2 (Csrp2), mRNA.	NM_007792	-2.6
Crip2	cysteine rich protein 2 (Crip2), mRNA.	NM_024223	2.5

**ECM/cell adhesion**			
Lox	lysyl oxidase (Lox), mRNA.	NM_010728	-3.4
Timp1	tissue inhibitor of metalloproteinase 1 (Timp1), mRNA.	NM_011593	-3.2
Timp2	tissue inhibitor of metalloproteinase 2 (Timp2), mRNA.	NM_011594	-3.0
Col6a1	procollagen, type VI, alpha 1 (Col6a1), mRNA.	NM_009933	-2.8
Col5a1	procollagen, type V, alpha 1 (Col5a1), mRNA.	NM_015734	-2.5
Mmp2	matrix metalloproteinase 2 (Mmp2), mRNA.	NM_008610	-2.0
Ptprs	protein tyrosine phosphatase, receptor type, S (Ptprs), mRNA.	NM_011218	2.5
Mfge8	milk fat globule-EGF factor 8 protein (Mfge8), mRNA.	NM_008594	2.6

**Cell migration**			
Ryk	receptor-like tyrosine kinase (Ryk), mRNA.	NM_013649	-2.7
Rtn4	reticulon 4 (Rtn4), transcript variant 5, mRNA.	NM_194054	-2.7
Axl	AXL receptor tyrosine kinase (Axl), mRNA.	NM_009465	-2.6
Wisp1	WNT1 inducible signaling pathway protein 1 (Wisp1), mRNA.	NM_018865	-2.5
Ccl7	chemokine (C-C motif) ligand 7	NM_013654	-2.4
Cyb5	cytochrome b-5 (Cyb5), mRNA	NM_025797	-2.3
Nup62	nucleoporin 62 (Nup62), mRNA.	NM_053074	2.3

**Others**			
Anxa5	annexin A5 (Anxa5), mRNA.	NM_009673	-3.0
Sumo1	SMT3 suppressor of mif two 3 homolog 1 (yeast)	NM_009460	-3.0
Sms	spermine synthase (Sms), mRNA.	NM_009214	-3.0
Cenpa	centromere autoantigen A (Cenpa), mRNA.	NM_007681	-2.9
Plec1	plectin 1 (Plec1), transcript variant 1, mRNA.	NM_011117	-2.9
Lmna	lamin A (Lmna), mRNA.	NM_019390	-2.9
Nedd8	neural precursor cell expressed, developmentally down-regulated gene 8 (Nedd8), mRNA.	NM_008683	-2.8
Pfdn5	prefoldin 5 (Pfdn5), mRNA.	NM_020031	-2.7
Rab2	RAB2, member RAS oncogene family (Rab2), mRNA.	NM_021518	-2.7
Fscn1	fascin homolog 1, actin bundling protein (Strongylocentrotus) purpuratus) (Fscn1), mRNA.	NM_007984	-2.6
Serpinb6a	serine (or cysteine) proteinase inhibitor, clade B, member 6a (Serpinb6a), mRNA.	NM_009254	-2.6
S100a1	S100 calcium binding protein A1 (S100a1), mRNA.	NM_011309	-2.5
Timm8a	translocase of inner mitochondrial membrane 8 homolog a (yeast)	NM_013898	-2.3
Ppap2a	phosphatidic acid phosphatase 2a (Ppap2a), mRNA.	NM_008903	-2.2
Fez2	fasciculation and elongation protein zeta 2 (zygin II)	NM_199448	-2.0
Ankrd1	ankyrin repeat domain 1 (cardiac muscle) (Ankrd1), mRNA.	NM_013468	1.7
Pole	polymerase (DNA directed), epsilon (Pole), mRNA.	NM_011132	1.9
Naglu	alpha-N-acetylglucosaminidase (Sanfilippo disease IIIB) (Naglu), mRNA.	NM_013792	2.0
Ruvbl2	RuvB-like protein 2 (Ruvbl2), mRNA.	NM_011304	2.0
Gig1	glucocorticoid induced gene 1 (Gig1), mRNA.	NM_133218	2.1
Soat1	sterol O-acyltransferase 1 (Soat1), mRNA.	NM_009230	2.1
Ris2	retroviral integration site 2 (Ris2), mRNA.	NM_026014	2.8
Ier3	immediate early response 3 (Ier3), mRNA.	NM_133662	2.8

Microarray analysis (Illumina Ref-8 BeadChip) was performed on GN-11 pSUPER-empty and AP-2α-low-expressing (α4-c) clones in duplicate and 510 modulated genes were found (305 decreased, 205 increased, see Methods). A selection of the most interesting modulated genes is shown. FC = Fold change.

**Table 2:** Common genes for GN-11 neurons and mouse embryo fibroblasts (MEFs)

**Gene**	**Description**	**GN-11**	**MEFs**
Lox	lysyl oxidase	-3.4	-1.6
Ccnd1	cyclin D1	-3.0	-1.5
Sms	spermine synthase	-3.0	1.6
Timp2	tissue inhibitor of metalloproteinase 2	-3.0	-1.7
Cenpa	centromere autoantigen A	-2.9	2.3
Birc5	baculoviral IAP repeat-containing 5	-2.6	2.1
S100a1	S100 calcium binding protein A1	-2.5	-1.6
Col5a1	procollagen, type V, alpha 1	-2.5	-1.5
Ccl7	chemokine (C-C motif) ligand 7	-2.4	1.6
Cdc2a	cell division cycle 2 homolog A (S. pombe)	-2.4	1.8
Timm8a	translocase of inner mitochondrial membrane 8 homolog a (yeast)	-2.3	1.7
Skp2	S-phase kinase-associated protein 2 (p45)	-2.2	1.7
Ppap2a	phosphatidic acid phosphatase 2a	-2.2	-1.7
Mmp2	matrix metalloproteinase 2	-2.0	-1.6
Fez2	Mus musculus fasciculation and elongation protein zeta 2 (zygin II) (Fez2), mRNA [NM_199448]	-2.0	1.6
Ankrd1	ankyrin repeat domain 1 (cardiac muscle)	1.7	2.1
Pole	polymerase (DNA directed), epsilon	1.9	1.8
Naglu	alpha-N-acetylglucosaminidase (Sanfilippo disease IIIB)	2.0	-1.8
Ruvbl2	RuvB-like protein 2	2.0	1.7
Gig1	Mus musculus glucocorticoid induced gene 1 (Gig1), mRNA.	2.1	-1.9
Soat1	sterol O-acyltransferase 1	2.1	-1.7
Pa2g4	proliferation-associated 2G4	2.2	1.7
Stmn1	stathmin 1	2.3	1.7
Tgif	TG interacting factor	2.4	2.5
Ris2	retroviral integration site 2	2.8	1.7
Ier3	immediate early response 3	2.8	1.5

The gene lists obtained from GN-11 neurons and mouse embryo fibroblasts microarray analyses were compared and an overlapping group of genes was identified which is shown here. FC = Fold change.

### Ingenuity Pathway Analysis of the newly identified AP-2α-regulated genes

**Figure 4:**
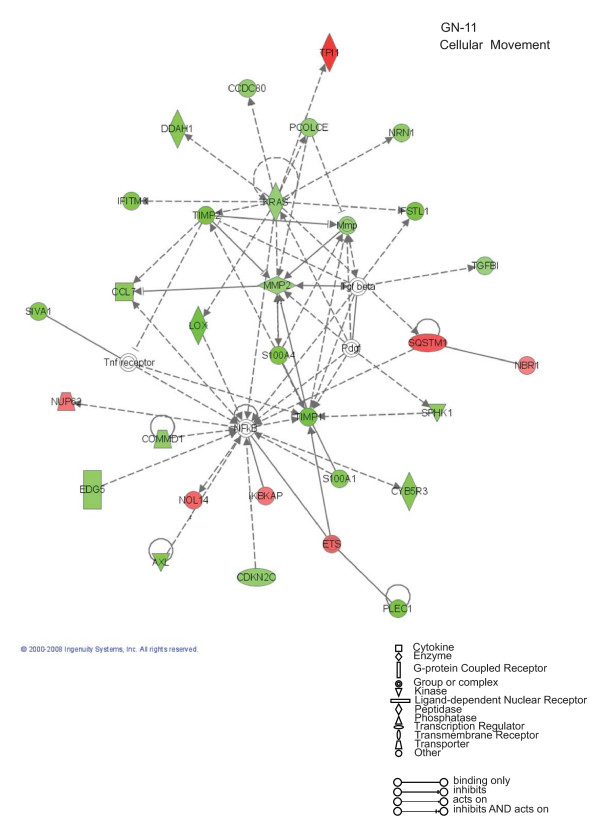
**Cellular Movement: Ingenuity Pathway Analysis for GN-11 neurons**. Specific functional networks of the newly identified AP-2α-regulated genes were obtained using Ingenuity Pathway Analysis systems and "cellular movement" is one of the two main networks found. Gene products are represented as nodes and biological relationships between two nodes as a line. Continuous lines indicate direct interactions, while dashed lines represent indirect connections. Shapes of nodes symbolize functional classes of gene products (see the figure legend). The green and red symbols represent down- and up-regulations, respectively, while the white symbols indicate genes absent in the dataset but related with the dataset genes.

**Figure 5:**
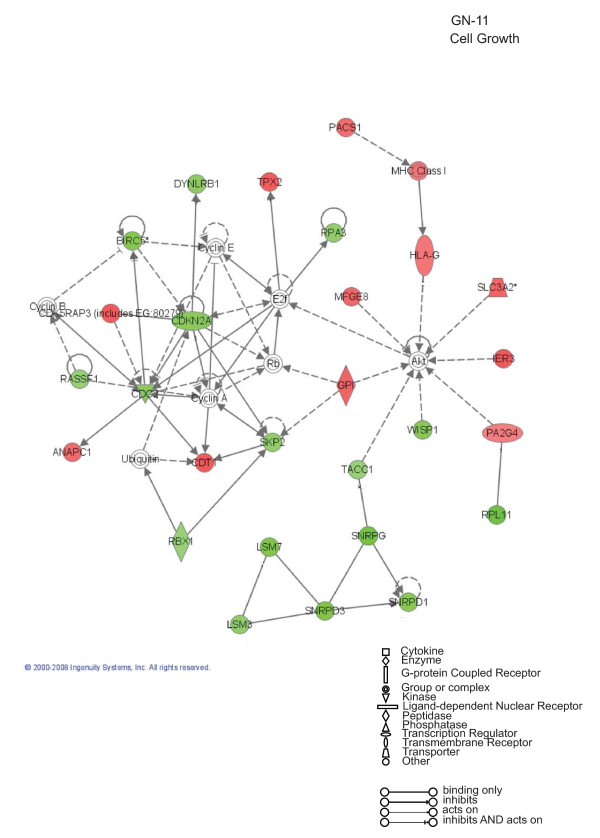
**Cell Growth: Ingenuity Pathway Analysis for GN-11 neurons**. Specific functional networks of the newly identified AP-2α-regulated genes were obtained using Ingenuity Pathway Analysis systems and "cell growth" is one of the two main networks found. Gene products are represented as nodes and biological relationships between two nodes as a line. Continuous lines indicate direct interactions, while dashed lines represent indirect connections. Shapes of nodes symbolize functional classes of gene products (see the figure legend). The green and red symbols represent down- and up-regulations, respectively, while the white symbols indicate genes absent in the dataset but related with the dataset genes.

**Figure 6:**
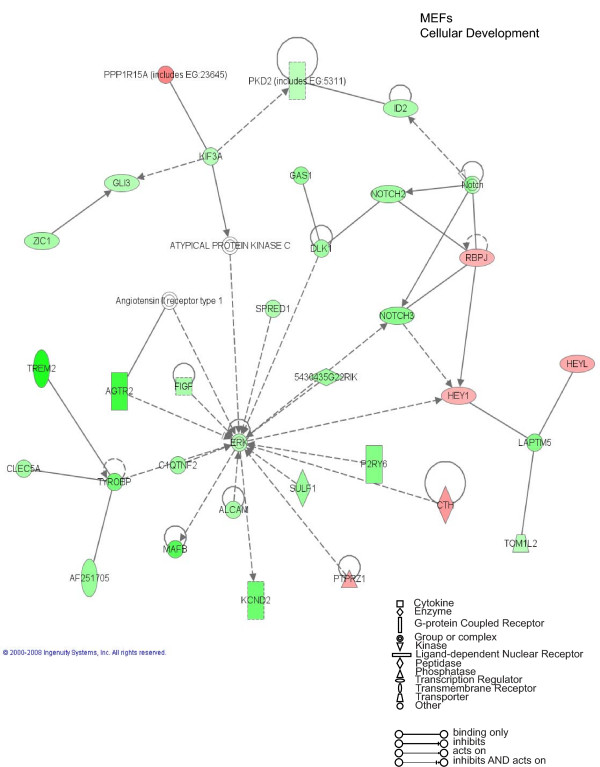
**Cellular Development: Ingenuity Pathway Analysis for mouse embryo fibroblasts (MEFs)**. Specific functional networks of the newly identified AP-2α-regulated genes were obtained using Ingenuity Pathway Analysis systems and "cellular development" is one of the two main networks found. Gene products are represented as nodes and biological relationships between two nodes as a line. Continuous lines indicate direct interactions, while dashed lines represent indirect connections. Shapes of nodes symbolize functional classes of gene products (see the figure legend). The green and red symbols represent down- and up-regulation, respectively, while the white symbols indicate genes absent in the dataset but related with the dataset genes.

**Figure 7:**
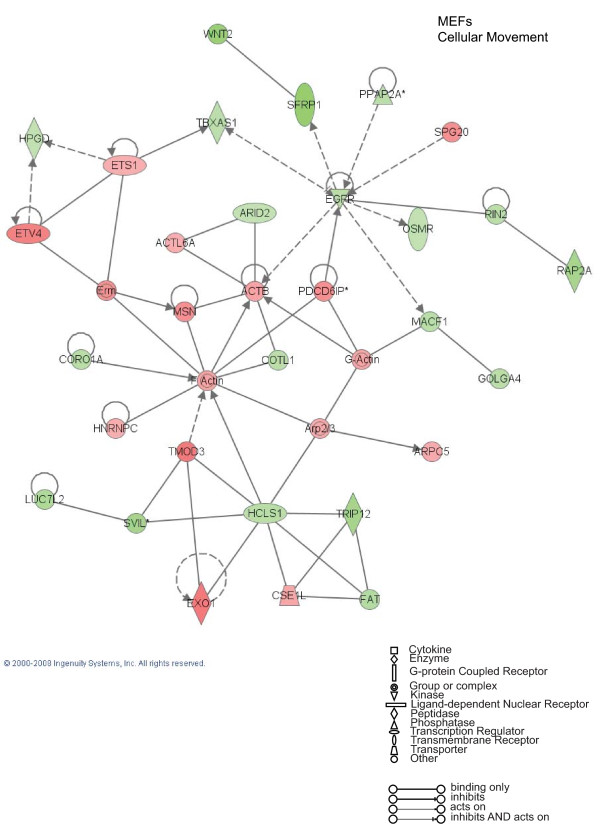
**Cellular Movement: Ingenuity Pathway Analysis for mouse embryo fibroblasts (MEFs)**. Specific functional networks of the newly identified AP-2α-regulated genes were obtained using Ingenuity Pathway Analysis systems and "cellular movement" is one of the two main networks found. Gene products are represented as nodes and biological relationships between two nodes as a line. Continuous lines indicate direct interactions, while dashed lines represent indirect connections. Shapes of nodes symbolize functional classes of gene products (see the figure legend). The green and red symbols represent down- and up-regulation, respectively, while the white symbols indicate genes absent in the dataset but related with the dataset genes.

To further evaluate the functional pathways in which the newly identified AP-2α-regulated genes are involved in GN-11 neurons and in MEFs we used the Ingenuity Pathway Analysis (IPA) system. Two main molecular networks were identified for GN-11 neurons (Figures 4 and 5). The first network includes 29 genes associated with cellular movement (Figure 4), for instance tissue inhibitors of metalloproteinases (*Timp1 *and *Timp2*), metalloproteinases (*Mmp2*), the receptor tyrosine kinase Axl, the chemokine (C-C motif) ligand 7 (*Ccl7*) and the cytochrome b5 reductase 3 (*Cyb5r3*, also known as diaphorase-1). The second network is associated with cell growth and includes 27 genes (Figure 5), such as CDK5 regulatory subunit associated protein 3 (*Cdk5rap3*), cyclin-dependent kinase inhibitor 2A (*Cdkn2a*) and WNT1 inducible signalling pathway protein 1 (*Wisp1*). These results indicate that AP-2α first of all regulates a panel of genes involved in GN-11 migration and proliferation. When we performed the same analysis on the MEF gene datasets two main molecular networks were identified (Figures 6 and 7). The first network is associated with cellular development and includes 31 genes such as Notch gene homologues 2 and 3 (*Notch2 *and *Notch3*), c-fos induced growth factor (*Figf*) and growth arrest specific-1 (*Gas1*) (Figure 6). The second network includes 31 genes associated with cellular movement, for instance epidermal growth factor receptor (*Egfr*), wingless-related MMTV integration site 2 (*Wnt2*) and secreted frizzled-related protein 1 (*Srfp1*) (Figure 7). For both cellular systems we identified a main network involved in cellular movement, however distinct pathways are activated.

### AP-2α binds to the Axl promoter and regulates its transcription

Among the genes we identified as AP-2α regulated in GN-11 neurons, one in particular came to our attention, the *Axl *gene, since it has been shown to control cell movement in GnRH^+ ^neurons [20,21]. Therefore, we studied its transcriptional regulation in more detail: we extracted its 5' genomic sequence (ENSMUSG00000002602) from the ENSEMBL bank database [22] and used 1 kb region upstream of the TSS to look for AP-2α; binding sites using the canonical AP-2α; positional weight matrix (PWM).

**Figure 8:**
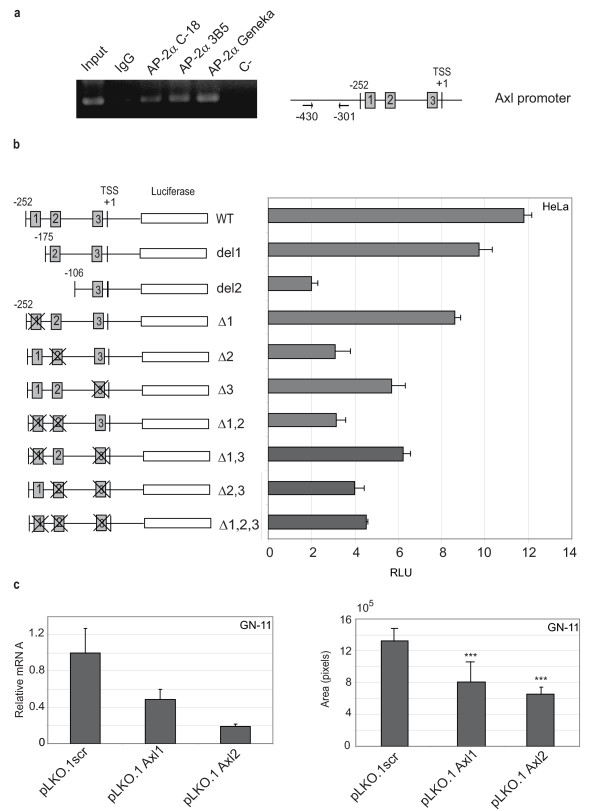
**AP-2α regulates GN-11 neuron migration via Axl**. (a) Three AP-2α binding sites were identified in the regulatory region (-1000/+1, +1 is the transcription start site (TSS)) of the mouse *Axl *gene (site 1 = GCCCCAAGG; site 2 = GCCAGGGGC; site 3 = GCCCAGGGG) and verified by chromatin immunoprecipitation (ChIP) using two primers at the indicated positions. Chromatin from GN-11 cells was cross-linked to proteins, extracted and immunoprecipitated with either AP-2α Abs (C-18 or 3B5 or Geneka) or non-specific IgG (negative control). DNA was analysed by polymerase chain reaction (PCR), using primers flanking the AP-2α putative binding sites in the *Axl *promoter. Input: not immunoprecipitated DNA. The experiment was repeated three times and one representative experiment is shown. (b) Schematic representations of the mouse *Axl *promoter fragments cloned into the pGL3-basic vector are shown (left panel). HeLa cells were transiently transfected with the various constructs together with a Renilla normalization vector (pRL-TK) and 48 hours later, luciferase activity was measured and normalized against Renilla activity. The experiment was performed in triplicate and repeated two or three times. One representative experiment is shown (right panel). RLU = relative luciferase units. (c) The effect of Axl down-regulation on cell movement was assessed for lentivirus-infected GN-11 neurons expressing either pLKO.1scr (scrambled) or pLKO.1Axl1 or pLKO.1Axl2. Axl down-modulation was evaluated by quantitative real-time PCR (left panel) while transwell migration (right panel) was analysed by plating the cells in serum-free medium in the upper chamber and allowing the cells to migrate over 10% foetal calf serum (FCS) medium for 18 hours through a porous membrane. The area occupied by migrating cells is shown. The experiments were performed in triplicate and repeated twice. Representative experiments are shown. The differences were statistically significant as measured by a two-tailed Student's *t*-test (***, *p *< 0.05). (In (b) and (c) the bars represent ± standard deviations.)

Three high score AP-2α binding sites were identified (site 1 = GCCCCAAGG; site 2 = GCCAGGGGC; site 3 = GCCCAGGGG). AP-2α binding was confirmed by chromatin immunoprecipitation (ChIP) analysis in the region including the three high score sites, as shown in Figure 8a, using three different anti-AP-2α antibodies. In order to test the function of the different AP-2α binding sites, 5' portions of the *Axl *gene were cloned into a luciferase reporter plasmid to generate pGL3-WT (WT), pGL3-del1 (del1) and pGL3-del2 (del2) vectors. The WT vector was further mutated at the AP-2α binding site 1 and/or 2 and/or 3 (seven-nucleotide deletions at the 5' end of the binding sites) to generate Δ1; Δ2; Δ3; Δ1,2; Δ1,3; Δ2,3; Δ1,2,3 vectors, as shown in Figure 8b, left panel. These vectors were transiently transfected in the human or mouse HeLa, 293T, NIH3T3 and NSC-34 cell lines, which are known to express the *AP-2α *and *Axl *genes. The results obtained with HeLa cells are shown in Figure 8b, right panel. The WT construct, which contains three intact AP-2α binding sites, showed the highest luciferase activity compared with del1 and del2 suggesting that this portion corresponds to the minimal promoter. Deletion of the region including AP-2α binding site 1 (del1) or 1 and 2 (del2) led to a 20% and 80% reduction in reporter activity, respectively, compared with WT.

Small deletions in the various AP-2α binding sites negatively modulated the luciferase reporter activity with different intensities depending on which or how many binding sites were mutated. When site 2 was mutated the most dramatic effect was observed suggesting that this site is the most responsive to AP-2α. However, reporter activity modulations occurred following mutations of any binding site which indicates that they are all functional. Similar results were obtained when various constructs were transfected in NIH3T3 (fibroblasts) or NSC-34 (neurons) mouse cell lines (see Additional file 3) suggesting that this is a general regulation which does not depend on the cell type or species used. The direct response of the WT construct to AP-2α was tested in HeLa cells following transfection of pAP-2α shRNA2 (for AP-2α silencing), pSP(RSV)AP-2α (for AP-2α overexpression) or control empty vectors [23] and reduced or increased luciferase activity were respectively observed (data not shown) as further proof of AP-2α-dependent transcriptional regulation of the *Axl *gene.

### AP-2α regulates GN-11 migration via Axl receptor tyrosine kinase (Axl)

To functionally investigate the role of Axl in GN-11 cell migration, these neurons were transduced with the lentivirus vectors pLKO.1scr, pLKO.1Axl1 or pLKO.1Axl2 in order to obtain stable expression of either scrambled or specific Axl shRNAs. High (70–80% reduction) and specific *Axl *gene silencing was obtained as measured by qRT-PCR (Figure 8c, left panel). Migration in response to serum was analysed in transwell assays and a decreased number of migrating cells (40–50% reduction) was observed following Axl knock down suggesting a direct involvement of *Axl *in cell migration (Figure 8c, right panel) as a single gene or in cooperation with the other modulated genes identified by microarray analysis. It is interesting to note that the reduction of migration was of the same magnitude as after knocking down AP-2α suggesting that AP-2α exerts its effects on migration mainly via Axl.

## Discussion

Our work presents a direct connection between the transcription factor AP-2α and the migration of the murine GN-11 cells, gonadotropin-releasing hormone (GnRH^+^) immortalized neurons, which show high migratory activity. In the organism GnRH^+ ^neurons migrate from the olfactory placode to the developing forebrain, to the septum and preoptic area of the hypothalamus and this migration is fundamental for the development of the normal reproductive functions [24]. For instance, in Kallmann's syndrome patients, olfactory axon development and migration of GnRH^+ ^neurons are impaired and this leads to a complex molecular pathogenesis which includes hypogonodotropic hypogonadism. This phenotypic alteration is secondary to a deficiency of GnRH in the hypothalamus with consequent lack of release of the pituitary gonadotropin luteinizing hormone (LH) and follicle stimulating hormone (FSH), involved in gonadal maturation [25]. Understanding the molecular mechanisms coordinated by AP-2α in GN-11 neuron migration can elucidate the role of AP-2 in the control of reproduction. No data are available for a possible role of AP-2α in reproduction since the knock-out models die perinatally [6,7,26]. By knocking down AP-2α, the only AP-2 isoform present in GN-11 cells, we demonstrated that AP-2α controls cell proliferation and cell movement via the activation of sets of specific genes interconnected with each other as revealed by microarray analysis and IPA. In particular, we demonstrated that migration depends on adhesion related kinase (*Axl*) gene expression induced by AP-2α following direct binding of this transcription factor to the canonical AP-2 binding sites present on Axl promoter.

AP-2 proteins are known to play relevant roles in neuron gene expression regulation and nervous system development [2,3]. Their expression coincides with specific developmental programmes such as the formation of the neural crest or the neural tube closure as demonstrated by AP-2α ablation in zebrafish [4] and in mouse [6,7,26]. Down-regulation of AP-2α in GN-11 neurons induces increased proliferation and the modulation of several genes known to be involved in cell cycle control such as cyclin D1 (*Ccdn1*), *Cdkn2a*, cell division cycle 2 homologue A (*Cdc2a*) and *Wisp1*. Interestingly, by comparing the microarray results obtained from GN-11 neurons and MEFs analyses we observed opposite modulations for genes involved in proliferation (i.e. *Cdc2a, Skp2*). These discrepancies could explain why the AP-2α-dependent modulation of proliferation we observed for GN-11 neurons was opposite to what was found for MEFs by Pfisterer et al. [27] suggesting that AP-2α is able to modulate the same genes in opposite directions depending on the cell context. However, our results are in line with previous AP-2α overexpression experiments which have shown induction of p21^WAF/CIP ^and inhibition of DNA synthesis and colony formation in various cell systems [28,29]. The direct mechanism of migration regulation by AP-2α in GnRH^+ ^neurons found in our work is perfectly in agreement with the observations presented previously in [19]. Kramer et al. revealed a decreased number of LHRH neurons in the brain of AP-2α-null mice at E13.5–E14.5 correlating with normal onset of AP-2α expression in LHRH neurons as they entered the central nervous system. One of the possibilities the authors suggested, among several, was that loss of AP-2α affected movement and migration of LHRH neurons to their appropriate target sites, however no molecular mechanism was presented at that time. From our microarray analysis in AP-2α silenced GN-11 neurons and AP-2α knock-out MEFs we learned that a large group of genes, much larger than we hypothesized, involved in cell growth and/or cell movement is modulated by AP-2α. Most of these genes were not known to be AP-2α direct or indirect targets therefore our investigation was essential in identifying gene expression profiles as well as the signal transduction pathways in which these genes operate. Known AP-2α regulated genes were identified correctly, i.e. *Mmp2 *(see [30]).

Some modulated genes involved in GN-11 neuron migration were tissue inhibitors of metalloproteinases (*Timp1 *and *Timp2*), metalloproteinase 2 (*Mmp2*), stathmin 1 (*Stmn1*), procollagens (*Col5a1 *and *Col6a1*), *Wisp1 *and the receptor tyrosine kinase *Axl*. In AP-2α null MEFs, epidermal growth factor receptor (*Egfr*), wingless-related MMTV integration site 2 (*Wnt2*), coronin actin binding protein 1A (*Coro1a*) and secreted frizzled-related protein 1 (*Sfrp1*) genes turned out to be regulated. It is important to observe that the genetic programmes activated seem to be cell-type dependent. Since only a few genes, i.e. *Timps*, *Mmps*, *procollagens *and *stathmin*, turned out to be modulated in both GN-11 cells and MEFs. The fact that AP-2α silencing in GN-11 neurons or in HeLa cells [23] or AP-2α ablation in MEFs led to decreased cell movement demonstrates that motility is depending on AP-2α transcription modulation in all of the cell systems analysed, but via distinct gene expression programmes. The pathway connections which involve the various AP-2α regulated genes identified by IPA and specifically involved in cell movement are under investigation. In this work we focused, in particular, on the AP-2α-driven transcription for the *Axl *gene, in GN-11 neurons. It is known that this gene regulates GnRH^+ ^cell migration [20] and that it is differentially expressed in migratory GN-10 neuronal cells compared with the post-migratory GT1–7 cells [31].

Moreover, the ligand of Axl, the growth arrest specific factor-6 (Gas6), is able to stimulate lamellipodial extension, membrane ruffling and chemotaxis of immortalized GnRH^+ ^neuronal cells via the Axl receptor [20]. We demonstrated that Axl is involved in serum-induced migration of GN-11 neuronal cells since down-modulation of Axl expression led to decreased migration in agreement with the recent data obtained in [21]. We have presented data showing that the *Axl *promoter contains three AP-2α binding sites, which are all functional as demonstrated by the analysis of *Axl *transcription. However *Axl *was not regulated in AP-2α-null MEFs or in AP-2α-silenced HeLa cells [23]. This suggests that Axl plays a major role in regulating AP-2α-driven migration in GN-11 cells, although Axl-dependent cell movement has already been demonstrated for other kind of cells, i.e. breast tumour cells [32].

## Conclusion

In conclusion, we have shown that AP-2α regulates a large number of genes in cells expressing this transcription factor. However, the genetic programmes driven by AP-2α are different, depending on the cell type analysed. In particular, we proved that GN-11 neuron migration depends on Axl expression which is directly linked to the presence of AP-2α. It has been recently demonstrated that Axl and one of its family members, Tyro3, are able to modulate female reproduction by influencing GnRH^+ ^neuron survival and migration [21]. Based on these findings we can predict a connection between AP-2α, Axl and reproduction. Axl and AP-2α seem to co-localize in the various nuclei of the mouse adult brain analysed using the Allen Brain Atlas [33]. This final observation suggests a major role for Axl not only in GN-11 cells but generally in neurons that depend on AP-2α-driven transcription.

## Methods

### Cell culture

GN-11 [24], HeLa, NIH3T3, 293T and NSC-34 cell lines as well as MEFs were maintained in Dulbecco's Modified Eagle's Medium containing 10 mM Glutamax, 4.5 g/ml glucose and 1 mM sodium pyruvate (DMEM-Glutamax™, GIBCO Invitrogen Life Technologies, Carlsbad, CA), supplemented with 10% heat-inactivated FCS (Seromed, GmbH), 25 mM HEPES pH 7.4 (GIBCO Invitrogen Life Technologies, Carlsbad, CA) and 100 μg/ml gentamicin (GIBCO Invitrogen Life Technologies, Carlsbad, CA). MEFs were isolated as stated in [27].

### Reagents, antibodies and DNA constructs

Three siRNA sequences (α1, α2, α3) targeting the murine AP-2α (GeneBank Identification code: 31981461) mRNA have been identified with the 'RNA structure' software [34] and cloned in the pSilencer 1.0-U6 vector (Ambion, Austin, TX) at the EcoRI/ApaI restriction sites as shRNAs. Targeting sequences: (α1) 842-GATCCCGGGTATTAACATC-861, (α2) 880-GAAAGGCCCCGTGTCCCTG-899, (α3) 943-GGACAACCTCTTCGGCGGC-962. The resulting expression vectors have been named respectively pSilencer 1.0-U6-shRNA-α1, 1.0-U6-shRNA-α2 or 1.0-U6-shRNA-α3. A fourth siRNA targeting sequence (α4) (5'-AACATCCCAGATCAAACTGTA-3') was obtained from QIAGEN (Stanford, CA), cloned in the pSUPERretro.puro vector (OligoEngine, Seattle, WA) at the BglII/HindIII restriction sites and the vector named pSUPERretro.puro-shRNA-α4. The pIRES.puro2 (Clontech Laboratories, Mountain View, CA) vector was used to select stable clones. pLKO.1 lentivirus scrambled (Cat. No. SHC008) and Axl (Cat. No. TRCN0000023309 and TRCN0000023313) shRNA expression vectors were purchased from Sigma (Sigma Aldrich, St Louis, MO) and respectively named pLKO.1scr, pLKO.1Axl1 or pLKO.1Axl2. pAP-2α shRNA2 and pSP(RSV)AP-2α vectors [23] were used to silence or overexpress AP-2α in HeLa cells together with their corresponding empty vectors. Primary antibodies used: anti-AP-2α mAb 3B5 or pAb C-18 or pAb Geneka, anti-Actin pAb C-11; secondary antibodies used: goat anti-mouse IgG HRP-conjugated, donkey anti-goat IgG HRP-conjugated. All antibodies were from Santa Cruz Biotechnology (Santa Cruz, CA) or Geneka- Active Motif (Carlsbad, CA, USA) and used at the producer's suggested concentrations.

### Generation of stable GN-11 cell clones and MEFs and culture conditions

GN-11 stable clones were generated by plating 50000 cells/cm^2 ^in 6 cm dishes in DMEM with no antibiotics and transfecting them 24 hours later using Lipofectamine2000^® ^(Invitrogen Life Technologies, Carlsbad CA) according to the manufacturer's instructions. Next 9 μg of either the empty pSilencer 1.0-U6 or the pSilencer 1.0-U6-shRNA-α1, 1.0-U6-shRNA-α2 or 1.0-U6-shRNA-α3 vectors were cotransfected with 1 μg of the pIRES.puro2 vector. Alternatively, GN11 neurons were transfected with 10 μg of either the empty pSUPERretro.puro or the pSUPERretro.puro-shRNAα-4 vectors. Transfectants were selected by adding Puromycin (10 μg/ml, Sigma Aldrich, St Louis, MO) to the medium 48 hours later. Two weeks later resistant clones were picked, expanded under selection, frozen and analysed. The clones used were named pSil-empty, pSUPER-empty, α1-b, α1-c, α2-a, α4-b andα4-c where α_*n *_corresponds to the shRNA sequence expressed. AP-2α +/+, +/- or -/- MEFs were generated from various embryos as described in [27].

### RNA isolation and qRT-PCR

Total RNA was isolated from the various clones using the Concert Cytoplasmic Reagent (Invitrogen Life Technologies, Carlsbad, CA) according to the manufacturer's instructions. qRT-PCR and calculations were carried out as described in [35]. Gene-specific primer sequences and QuantiTect^® ^Primer Assay catalogue numbers are available on request.

### Immunoblotting

Total protein extracts were obtained using a boiling buffer containing 0.125 M Tris/HCl, pH 6.8, and 2.5% sodium dodecyl sulphate (SDS). Proteins (25 μg) were separated by SDS (12%) polyacrylamide gel electrophoresis (PAGE) and electroblotted on to polyvinylidene fluoride (PVDF) membranes (Bio-Rad, Hercules, CA). Membranes were blocked in 5% non-fat milk, tris buffered saline (TBS)-Tween buffer (137 mM NaCl, 20 mMTris/HCl, pH 7.6, 0.1% Tween-20) overnight at 4°C, then incubated with appropriate secondary antibodies for 1 hour at room temperature, and visualized by enhanced chemiluminescence (ECL^®^, Amersham Biosciences, Pisactaway, NJ). Anti-AP-2α mAb 3B5, anti Actin pAb C-11, goat anti-mouse IgG HRP-conjugated, donkey anti-goat IgG HRP-conjugated antibodies (all from Santa Cruz Biotechnology, Santa Cruz, CA) were used.

### Proliferation assay

The proliferation assay was performed according to the protocol described in [36]. Briefly, 5000 cells per well were plated in 96-well plates in complete medium. Cells were serum starved 24 hours. Complete medium was then added and cells were allowed to grow for 24, 48 or 72 hours. At different time points cells were fixed in 2% glutaraldehyde. Plates were stained with 0.1% crystal violet solution and bound dye was solubilized using 10% acetic acid. The optical density of the dye extracts was measured directly in plates using a Microplate Reader HTS (Perkin Elmer, Waltham, MA) at 590 nm wavelength.

### Motility assay

The wound healing motility assay was used to measure two dimensional movements. Cells were grown to confluency in six-well plates, serum starved for 24 hours, then a cross wound was made on the monolayer using a sterile 200 μl pipette tips. Cells were rinsed three times with phosphate buffered saline (PBS) and placed in either serum-free DMEM or 10% FCS-DMEM. Two-dimensional cell movements were quantitated by measuring the distance covered by the migrating cells. For each experiment the right arm near the cross was photographed. Photos were taken at *t *= 0 hours and at *t *= 24 hours using a LEICA DM IRB microscope equipped with a CCD camera Cool SNAPPro (Media Cybernetics, Silver Spring, MD). Images were edited with Image ProPlus software (Media Cybernetics, Silver Spring, MD). The two-dimensional movement of the cells was quantitated by measuring the surface area occupied by the migrated cells using the Scion Image 1.62 software [37,38].

### In vitro migration assay

10^5 ^GN-11 neurons or MEFs were resuspended in 200 μl serum-free DMEM and seeded in the upper chambers of 24-well Falcon cell culture inserts (BD Biosciences, NJ) over a porous polyethylene terephthalate membrane (8.0 μm pore size, 1×10^5 ^pores/cm^2^). The lower chamber was filled with either serum-free medium or DMEM plus 10% FCS. Plates were incubated at 37°C in 5% CO_2 _and the number of cells migrated to the lower side of the membrane was analysed 24 hours later. Here the cells were rinsed in PBS, fixed in 2% glutaraldehyde for 15 min at room temperature, washed five times in water, stained with 0.1% crystal violet for 20 min at room temperature, washed again five times in deionized water, air dried and photographed using a LEICA DM IRB microscope equipped with a CCD camera Cool SNAPPro (Media Cybernetics, Silver Spring, MD). Images were edited with Image ProPlus software (Media Cybernetics, Silver Spring, MD). The area occupied by the migrated cells was measured by using Scion Image 1.62 software [37,38].

### Microarray analysis

Microarray analysis of gene expression in response to AP-2α knock down in GN-11 cells was performed using the Illumina BeadChip system (Illumina, Inc, San Diego, CA). We used 500 ng of total RNA to obtain labelled, amplified cRNA for each sample to hybridize the Illumina Ref-8 BeadChips according to the manufacturer's instructions (Illumina, Inc, San Diego, CA). Arrays were scanned with an Illumina BeadArray Reader confocal scanner and data processed and analyzed using Illumina BeadStudio software (Illumina, Inc, San Diego, CA). Raw Illumina data were rank-invariant normalized with the BeadStudio software (Illumina, Inc, San Diego, CA), which was also used to assess differential expression between the α4-c clone and the control pSUPER-empty clone, based on two RNA preparations from each sample.

After normalization, genes were filtered by their 'detection' value, which had to be 0.99 (significantly detected) in at least one of the two samples. The filter was passed by 6658 genes. Subsequently, we identified differentially expressed genes using the Illumina custom error model implemented in BeadStudio, which provides an expression difference score ('DiffScore') taking into account background noise and sample variability [39]. We chose a DiffScore threshold of 20, corresponding to a p-value of 0.01, and thereby to a null hypothesis of about 66 genes passing the test by chance.

Indeed, 1220 genes passed the test, indicating a false discovery rate of about 5%. To restrict the analysis to the most regulated genes, an additional filtering criterion was that the average expression fold-change between the α4-c clone and the control pSUPER-empty clone had to be at least 1.5-fold, which lead to the identification of 510 modulated transcripts (305 decreased and 205 increased). Sample permutation analysis confirmed that under these conditions the false discovery rate was well below 5%. Microarray analysis for MEFs was carried on using the Agilent Whole Mouse Genome 44 K system (Agilent Technologies, Palo Alto, CA) as described previously [23,40]. Briefly, 750 ng of total RNA were used to obtain labelled cRNA for each sample to hybridize the arrays following Agilent's protocol. Then, slides were scanned, and images were analysed using Feature Extraction software version 7.6 (Agilent Technologies). Raw data files containing feature and background intensities and related statistical parameters were then loaded on the Resolver SE System (Rosetta Biosoftware), together with the scan images and the Agilent Mouse Whole Genome pattern file. Data processing and normalization were performed using the Agilent Mouse Whole Genome platform-specific error model and yielded, for each sequence on the array, an expression fold-change and a p-value to assess the statistical significance of its modulation in the samples compared with references. Three independent preparations of AP-2α +/- and -/- MEFs were analysed in our experiments. By choosing a p-value of 0.01 a false discovery rate of about 5% was calculated. Weighted average was applied to replicate measures. To restrict the analysis to the most regulated genes, an additional filtering criterion was that the average expression fold-change between the AP-2α -/- and the AP-2α +/- MEFs had to be at least 1.5-fold, which lead to the identification of 1492 modulated transcripts (878 decreased and 614 increased).

### IPA

The Ingenuity Pathways Knowledge Base [41] is currently the world's largest database of knowledge on biological networks, with annotation curated by experts. We exploited this database to define the presence of functional associations within the genes detected by microarray analysis and to identify differences between the ontological gene classes that were enriched among differentially expressed genes. This ontological gene classification provides the controlled vocabulary to describe gene and gene product attributes.

### Bioinformatic analysis of the Axl promoter

The *Axl *regulatory regions and the putative AP-2 binding sites were analysed by using the ENSEMBL bank database [22] and the canonical AP-2α PWM [42]. The Retrieval of non-coding Regulative Elements from annotated genome databases (RRE) software was used to design primer pairs to perform chromatin immunoprecipitation analysis for the *Axl *gene. RRE is available at [43] (see also [44]).

### ChIP assays

ChIP was performed using the ChIP-IT™ Kit (Active Motif, Carlsbad, CA) reagents and protocols. Primer pairs were designed using the RRE software [43,44] and sequences are available upon request.

### Database searches

GeneBank searches were performed using the BLAST service at the National Center for Biotechnology Information (NCBI) [45].

### Mouse Axl promoter cloning

A 5' fragment of the mouse *Axl *gene (500 base pairs) was obtained by PCR using GN-11 genomic DNA as template and specific primers (sequences are available from the author upon request). The PCR product was cloned into pCR^® ^2.1-TOPO vector (Invitrogen Life Technologies, Carlsbad, CA), sequenced, excised using KpnI and XhoI and subcloned in pGL3-basic vector (Promega, Madison, WI). 75 nucleotide-deletions of this fragment were obtained at its 5'end by convenient restriction enzyme digestions and WT, del1 and del2 constructs were generated. The WT vector was further mutated at the AP-2α binding site 1 and/or 2 and/or 3 to obtain seven-nucleotide deletions at the 5' end of each binding site by using the QuikChange Site-Directed Mutagenesis kit (Stratagene, La Jolla, CA) and Δ1; Δ2; Δ3; Δ1,2; Δ1,3; Δ2,3; Δ1,2,3 vectors were obtained.

### Transient transfections and luciferase assays

The 6 × 10^4 ^cells were seeded in 24-well plates 24 hours before transfection. Cells were then transfected using Lipofectamine 2000 (Invitrogen Life Technologies, Carlsbad, CA) with 500 ng of WT, del1 or del2 or Δ1 or Δ2 or Δ3 or Δ1,2 or Δ1,3 or Δ2,3 or Δ1,2,3 vectors together with 20 ng of pRL-TK plasmid to normalize for transfection efficiency (Promega, Madison, WI). After 48 hours following tranfection cell extracts were prepared by adding 100 μl of 1 × Passive Reporter Lysis Buffer (Promega, Madison, WI). Luciferase activity was measured using the Dual Luciferase Assay System (Promega, Madison, WI) according to the manufacturer's instructions. Each transfection was performed in triplicate and three independent experiments were carried on.

### Lentiviral infections and gene silencing with shRNAs

Lentiviruses were produced by 293T cells as reported in [23]. Supernatant was used to infect 2 × 10^5 ^cells in six-well plates in presence of 8 μg/ml Polybrene.

### Statistical analyses

Statistical significance of the various experiments was tested by performing an *F*-test followed by a two-tailed *t*-test. Here *p *indicates the probability of identity of the distributions.

## Abbreviations

*Ark*: Adhesion Related Kinase; ChIP: chromatin immunoprecipitation; DMEM: Dulbecco's Modified Eagle's Medium; ECM: extracellular matrix; FCS: foetal calf serum; FSH: follicle stimulating hormone; GnRH^+^: gonadotropin releasing hormone; GO: Gene Ontology; IPA: Ingenuity Pathway Analysis; LH: luteinizing hormone; LHRH: luteinizing hormone receptor; MEF: mouse embryo fibroblast; PAGE: polyacrylamide gel electrophoresis; PBS: phosphate buffered saline; PCR: polymerase chain reaction; PVDF: polyvinylidene fluoride; PWM: power weight matrix; qRT-PCR: quantitative real-time polymerase chain reaction; RNAi: RNA interference; SDS: sodium dodecyl sulphate; TBS: tris buffered saline.

## Authors' contributions

FO and DT conceived the project. DT supervised the research. FO performed the experiments. RJ and HS provided the AP-2α knock-out MEFs. RAC provided the IPA software. DT, MDB and PS supported the research.

## Supplementary Material

Additional file 1 **Table of differentially expressed genes in AP-2α low-expressing GN-11 clones**. Microarray analysis (Illumina Ref-8 BeadChip) was performed on the GN-11 pSUPER-empty and α4-c clones in duplicate. The complete list of 510 modulated genes (*p *< 0.01; fold change > 1.5) is shown. Click here for file

Additional file 2 **Table of differentially expressed genes in AP-2α -/- versus +/- mouse embryo fibroblasts (MEFs)**. Microarray analysis (Whole Mouse Genome Agilent 44 K) was performed on RNA obtained from three different preparations of AP-2α +/- or -/- mouse embryo fibroblasts. The complete list of 1492 modulated genes (*p *< 0.01; fold change > 1.5) is shown. Click here for file

Additional file 3 **Regulation of the *Axl *promoter by AP-2α**. Schematic representations of the mouse *Axl *promoter fragments cloned into the pGL3-basic vector are shown (left panel). (a) NSC-34 or (b) NIH3T3 cells were transiently transfected with the various constructs together with a Renilla normalization vector (pRL-TK) and 48 hours later luciferase activity was measured and normalized against Renilla activity. The experiment was performed in triplicate and repeated twice. One representative experiment is shown (right panel). RLU = relative luciferase units. Click here for file
